# Effect of Scan Strategies and Use of Support Structures on Surface Quality and Hardness of L-PBF AlSi10Mg Parts

**DOI:** 10.3390/ma13102248

**Published:** 2020-05-13

**Authors:** Ronny M. Gouveia, Francisco J. G. Silva, Eleonora Atzeni, Dušan Sormaz, Jorge Lino Alves, António Bastos Pereira

**Affiliations:** 1 ISEP—School of Engineering, Polytechnic of Porto, 4200-072 Porto, Portugal; ronnygouveia@gmail.com; 2 Dipartimento di Ingegneria Gestionale e della Produzione, Politecnico di Torino, 10129 Torino, Italy; eleonora.atzeni@polito.it; 3 Department of Industrial and Systems Engineering, The Ohio University, Athens, OH 45701, USA; sormaz@ohio.edu; 4 Faculty of Engineering, University of Porto, 4200-465 Porto, Portugal; falves@fe.up.pt; 5 TEMA—Centre for Mechanical Technology and Automation, Department of Mechanical Engineering, Campus de Santiago, University of Aveiro, 3810-193 Aveiro, Portugal; abastos@ua.pt

**Keywords:** additive manufacturing (AM), additive manufacturing materials, AlSi10Mg, microstructure, roughness, residual stress, process parameters, pattern strategies, laser powder bed fusion, L–PBF, Al–Mg oxides, hardness, selective laser melting (SLM)

## Abstract

Additive manufacturing allows for a great degree of design freedom and is rapidly becoming a mainstream manufacturing process. However, as in all manufacturing processes, it has its limitations and specificities. Equipping engineers with this knowledge allows for a higher degree of optimization, extracting the most out of this technology. Therefore, a specific part design was devised and created via L-PBF (Laser Powder Bed Fusion) using AlSi10Mg powder. Certain parameters were varied to identify the influence on material density, hardness, roughness, residual stress and microstructures. It was found that on heat treated parts laser pattern strategy is one of the most influential aspects, showing that chessboard and stripes 67° improved outcome; average R_a_ roughness varied between 8–12 µm, residual stress was higher on vertical surfaces than horizontal surfaces, with the combination of support structures and stripes 67° strategies generating the lowest residual stress (205 MPa on a lateral/vertical face), hardness was non-orientation dependent and larger on samples with chessboard fabrication strategies, while microstructures were composed of α–Al dendrites surrounded by Si particles. The distribution and grain size of the microstructure is dependent on location regarding melt pool and HAZ area. Furthermore, Al–Mg oxides were encountered on the surface, along with pores generating from lack of fusion.

## 1. Introduction

Additive Manufacturing (AM) is a recent manufacturing process that has created a disruption with traditional manufacturing methods commonly used up until a decade ago [1]. This technology started with the manufacture of prototypes (essentially using polymeric materials), but has quickly evolved into metals, allowing today the production of numerous components for very important sectors, such as the aeronautical industry [2], medical supplies [3] and consumer goods [4]. Essentially, it is a technique capable of building parts through the successive deposition of relatively thin layers, in a very diverse range of polymeric, metallic alloys or even composite materials. It allows the production of parts with complex geometries with minimal waste of material, thus, making it a sustainable process [5]. Due to the rapid growth and evolution that this technique has undergone, it was necessary to establish some standardization, having appeared in 2015 the first standard, ISO/ASTM 52900:2015 [6] which establishes the general principles of the process and terminology to be used, and which gave rise to other more specific standards within the same family. This standard established the nomenclature of Laser-based Powder Bed Fusion (L–PBF) for a process that was previously known as Selective Laser Melting (SLM), a name by which it is still known. However, there are still some problems that need to be overcome, namely the need to increase quality and reproducibility, which can be improved through algorithms that allow better control of the process and optimization of parameters [7,8].

Laser-based Powder Bed Fusion (L–PBF) as a manufacturing technology, has demonstrated its capacity to respond positively to the high demand for metal parts the market has generated in recent years. This technology allows the production of components with characteristics very similar to those exhibited by parts manufactured using conventional technology [9]. The L–PBF process consists of using a concentrated laser beam, which moves nimbly over a bed of powder of a given alloy, according to the path traced by the software that slices the three-dimensional (3D) model of the part to be built. A thin layer of powder is melted and cooled/solidified quickly, producing another layer of solid material that will constitute the final part [10]. The L–PBF technology has been used extensively to carry out numerous studies in different metallic alloys, such as Ti6Al4V [11,12], IN718 [13,14], AISI 316 L [15,16], and AlSi10Mg [17,18], among others [19].

Beyond Ti6Al4V, another alloy that stands out in terms of research is AlSi10Mg, as it is a light alloy with very interesting properties [20,21], such as high strength-to-weight ratio, low thermal expansion coefficient, among other. In addition, it was verified by Tang et al. [22] that the properties obtained in parts made by the SLM process had properties very similar to those obtained through conventional processes, such as high-pressure die-casting. Furthermore, when subjected to additional processing, such as machining or vibratory polishing, the fatigue behavior of this alloy can be improved, surpassing that of the common Al6061 alloy [23]. However, the parts obtained through AM exhibit anisotropy and properties that depend on the building direction [24,25]. The effects of strong anisotropy on thermophysical properties have also been reported by Strumza et al. [26], having observed significant differences in properties, such as the expansion coefficient, conductivity and thermal diffusivity, due to the fact that there is a preferential orientation and a lack of homogeneity in the distribution of the porosities, influenced by build direction (X or Z axis dependent). These effects are mainly due to the preferential orientation of the aluminum grains, the texture and the distribution of pores. Improvement in mechanical properties is one of the greatest concerns of researchers [27]. Mechanical properties are strongly conditioned by the alloy’s microstructure. It has been observed that the mechanical strength presented by AlSi10Mg alloy is based on the fine particle distribution of Si eutectic, due to the high cooling rate characteristics of the process [28,29]. One of the factors that tends to affect mechanical properties more significantly, both in terms of mechanical strength and fatigue resistance, are defects of lack of fusion, which translate into porosities. Kan et al. [27] have reported that the high hardness and mechanical strength exhibited by the AM AlSi10Mg alloy are due to nano-crystalline dendritic arrangements of Si particles whose dendrite arm spacing is normally less than 1 μm and is usually aligned along the building direction. In this work, the hardness obtained in the as-built samples was 140 HV. However, it decreased by about 20% when the samples were subjected to T6 type treatment. The yield strength and tensile strength reached 307, and 424 MPa, respectively, in the as-built state, values that also decreased after a T6 treatment to 225, and 337.5 MPa, respectively. On the other hand, the displayed ductility is extremely low, not exceeding 3%. In turn, Chen et al. [30] reported hardness values for this same alloy between 90–100 HV, being direction dependent. In another study by Takata et al. [31] it was verified that hardness decreased as a function of wall thickness, when using SLM, going from 112 HV in 10 mm thick walls, to 107 HV when measured in 0.3 mm thin walls. A similar study was carried out by Majeed et al. [32], also concluding that the hardness is higher in thick walls, with values of 137.3 HV for thicknesses of 5.0 mm, and 102.4 HV for 1.0 mm, based on L–PBF AlSi10Mg samples. Kan et al. [27] states that these lack-of-fusion defects tend to align in the direction perpendicular to the building direction. Depending on the direction in which the traction is carried out, if the lack-of-fusion defects are aligned with the load axis, the mechanical strength, exhibited by the material, is greater than if the porosities are aligned in the direction perpendicular to the load. The properties of the parts obtained in AlSi10Mg alloy depend strongly on the parameters selected for the process. In a study by Liu et al. [33], based on the Taguchi technique and analysis of variance (ANOVA), it was found that the parameter that exerts the most influence on the additively manufactured AlSi10Mg parts is laser power, being 49.43%, while scanning speed is the second most influential variable, with 33.74%. These authors also concluded that in order to obtain high densification, it is necessary to use a high energy density. However, Calignano et al. [34] in a study carried out based on the manufacture of thin walls in AlSi10Mg by L–PBF, concluded that the power density, by itself, is not a parameter reliable enough to predict the complexity represented by the formation and propagation of the melting bath in the process. Another important concept is volumetric energy constant (VEC), which essentially governs the density of energy per volume. Instead of solemnly focusing on laser power, VEC allows to generate similar fusion conditions while utilizing different laser powers, scan speeds and layer heights. Several authors have experimented with different ranges of VEC and have achieved results of 99% density. VEC is of course material dependent, but with regards to AlSi10Mg Wang, et al. [35], Casati, et al. [36] and Aboulkhair et al. [29] determined that VEC levels proximate to 200 J/mm^3^ allowed to achieved densities of above 99%. However, as VEC is composed of several important parameters, similar values of VEC can result in different outcomes [37]. Another interesting concept is energy per layer, which is dependent on the layer area and build direction and can vary during a print even when the VEC is kept constant [25,38]. 

Residual stresses imposed by the L–PBF process on AlSi10Mg alloys have also been subject of study. Samples produced by this process and subsequently annealed for stress relief at 310 °C for 1 h, followed by a mechanical shot-peening treatment, revealed that the surface presents compressive stresses that reach their maximum at around 0.1 mm of depth, and that were diluted in function of the depth, canceling out at a depth of about 0.7 mm from the surface. In any case, the stresses measured through the hole-drilling method never exceeded 100 MPa [39]. It is worthy to note that the geometry of the parts to be built, as well as the use and positioning of the supports, are decisive factors in the installation of residual stresses in the components produced through L–PBF with AlSi10Mg. On the other hand, a study carried out by Leary et al. [40] with the aim of assessing the mechanical resistance offered by the supports and their role in the flow of heat generated locally by the process, allowed to verify that the mechanical resistance offered by the supports is influenced by its height, and that the proximity of different supports allows increased heat dissipation and load bearing capacity. An analysis of the failure mechanisms showed that ductile behavior was observed. Several authors have investigated the effects of different heat treatments in AM AlSi10Mg parts, with temperatures ranges and holding times varying. For stress relief, it has been found that heat treatments of 300 °C for 2 h show good results, and in some cases temperatures as low as 200 °C were able to produce positive results [41,42,43]. However, other authors have studied the effects of conventional heat treatments such as T6 annealing and have determined that this treatment, followed by water quenching and aging treatments allow to modify the microstructure, rearranging the fine α–Al cells and segregated Si, eliminating any trace of laser tracks and ultimately showing coarser grains and globular particles of Si [27,44,45,46]. The drawbacks brought by the use of high temperature treatments is that important mechanical properties such hardness and strength diminish when compared to the as-built values. Mechanical properties, such as hardness have also received research attention, indicating that this property varies based on part orientation, thickness and post heat treatment condition [32,47,48].

The characteristic deformations of the process in complex shaped parts have also been widely discussed. Han et al. [49] studied deformation in overhang structures, both in full-circle and half-circle formats, concluding that diameters greater than 15 mm require the use of supports. It was found that the puddle melted in the dross-free zone is smaller than that existing in the dross zone, where the melting pools were supported by the solidified layers. This difference is directly related to the difference in thermal conductivity between the already solidified layers and the layers with bulk powder. 

Depending on the application, surface roughness is an important characteristic and dictates the interaction between two surfaces. The ability to produce final parts with low surface roughness is highly desirable in AM, as it may, in some cases, avoid the need for additional manufacturing processes. Surface roughness is highly influenced by the chosen AM process and the selected production parameters, which can generate surface defects and changes in surface morphology [50], for instance, defects caused by balling, which are droplets of molten material with a diameter larger than the laser spot diameter that spread out through the surface. This phenomenon is caused by a lack of wettability originating from dissimilar thermal grades in the melt pool, hindering melt pool adhesion to previous layers/passes/substrate, breaking up the laser melt path into large balls of material [51]. Satellites are another type of defect that occur when insufficient power is present, giving way to partially fused powder particles that retain an irregular shape and protrude from the surface. Particles in the periphery of the laser beam are also at risk of becoming satellites by partial fusing and sticking to the melt pool [29,52]. A lack of fusion and porosity are also defects that lead to higher surface roughness. Part orientation and inclination is also highly influential on surface roughness as the effect of gravity on the liquid melt pool must be taken into account [39]. Dimensional and shape precision was also explored by Calignano et al. [53] in the production of complex interior channels, using L–PBF AlSi10Mg alloy, concluding that final surface roughness and part geometry fidelity, in relation to the 3D model, depends strongly on the parameters of the conversion of the model to an STL file, the selected orientation and the parameters chosen for the process. The values usually suggested as maximum for the eccentricity control of the STL file are 1/20th of the layer thickness, but the values should always be greater than 1 μm. For an interior radius of 30 mm, a maximum deviation of 0.023 mm was obtained, regarding the original 3D model. In order to improve the fidelity of the geometry of components produced by L–PBF in AlSi10Mg alloy, Yeung et al. [54] developed an algorithm capable of controlling the power supplied to the laser system at each location, thus, modulating the deposition process according to the location where the beam is based on a parameter called the Geometric Conductance Factor, and lowering the laser power when the beam is close to edges or overhangs. This algorithm however, implies a new redefinition of the parameters for the entire process.

The structure of this article is as follows: Section 2 discusses the materials and methods utilized to achieve experimental samples and data; Section 3 states the experimental results regarding the weight, residual stress, surface roughness, hardness and microstructure retrieved, as well as their discussion; concluding remarks are made in Section 4.

## 2. Materials and Methods 

### 2.1. Materials, Equipment and Process Parameters

The material chosen to be tested in this work is an AlSi10Mg aluminum powder specific to L–PBF processes, being a very common build material in the AM industry and used across many brands of PBF machines. Specifically, the material used is commercially known as CL31AL and manufactured by Concept Laser GmbH (Lichtenfels, Germany). The chemical composition and common properties of AlSi10Mg can be seen in Table 1. 

The L–PBF equipment used to produce the samples was a Mlab Cusing R manufactured by Concept Laser GmbH (Lichtenfels, Germany), with a build envelope of 90 × 90 × 80 (mm^3^) (x,y,z). The raw alloy powder was sieved prior to production in order to ensure a granulometry size smaller than 50 µm. The chosen machine parameters can be seen in Table 2. Process parameters were selected in order to maintain a volume energy constant (VEC) of $E=195Ws/mm^{3}$, being defined by Equation (1) [60],

(1) $$E=\frac{P}{v\times D_{s}\times h_{s}}$$

where E is volume energy constant; P is laser power (W); v is scanning speed (mm/s); *D_s_* the hatching distance (mm) and *h_s_* is layer thickness (mm). This value of VEC is on par with other studies which have researched the L–PBF of AlSi10Mg, having achieved parts with relative densities of 99% and higher at these levels of VEC [29,35,36].

Several sets of samples were produced (3 samples per set) with the parameters, shown in Table 2. In order to optimize print area and minimize costs, print jobs had multiple sets of samples at once, with sample set 1, 2 and 3 being printed during the same jobs while samples set 4, 5 and 6 were printed together in other jobs. Part placement in the chamber was done to guarantee equal distribution and gas flow, as well as ensure a 5° angle relative to the re-coater, thus minimizing forces between the re-coater and side walls. The process parameters designated in Sample_Set_01 and Sample_Set_04 are considered by the equipment manufacturer as the generalist recommended settings for the fabrication of parts with AlSi10Mg, being the difference between these sets using build supports. Besides the use of supports, the other parameters that were varied were build pattern strategies and layer thickness.

**Figure 1:**
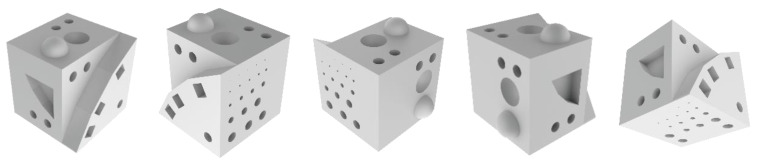
CAD Illustration of Sample to be Manufactured. Views of its different faces.

Figure 1 shows a CAD (Computer-Aided Design) representation of the experimental sample. The specificities of the sample designs will not be discussed in this paper, as it is part of a bigger research project, which addresses further aspects of metal AM. When employed, build supports were generated using Materialise Magics software (Materialise, Leuven, Belgium), while the slicing/g-code was generated using Materialise Build Processor software (Materialise, Leuven, Belgium). After the production process was completed, the samples were removed from the build plate using an electrical discharge machining (EDM), cleaned of any support build structures and heat-treated for 1 h at 310 °C. Each sample set is composed of three samples, generating a total of 18 produced samples. 

### 2.2. Sample Evaluation Equipment 

Several different properties/aspects of the produced samples were evaluated using a wide variety of equipment. Table 3 summarizes the properties and equipment used to analyze the samples.

**Figure 2:**
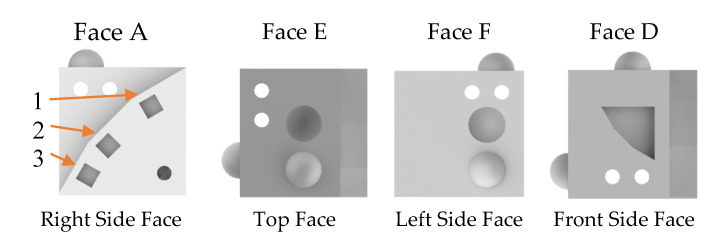
Sample Faces Subjected to Roughness Measurement.

Firstly, all samples were weighed using a Denver Instrument APX–200 (Denver Instruments, NY, USA) scale. Secondly, the roughness of the outer surface of the samples was determined using a Mahr Perthometer M2 (Mahr, Göttingen, Germany) device. Five distinct areas were chosen to be measured. Using Figure 1 and Figure 2 as a guide, these areas were:

- The 30° inclined ramp (face A–1)
- The 45° inclined ramp (face A–2)
- The 60° inclined ramp (face A–3)
- The top side face (face E)
- The left side face (face F)

Thirdly, residual stress was analyzed with a Rigaku Smartlab^®^ X–Ray Diffractometer (Rigaku, Wilmington, MA, USA), equipped with a CuK_α_ radiation source. According to the equipment manufacturer, the analysis of XRD peaks should be made at the highest *2θ* angle possible that presents a significant peak (following the Bragg-Brentano diffractometer principle). At high *2θ* angles crystalline structures show the effects of residual stress more easily [61]. Poisson’s ratio and Young’s modulus were set to 0.33 and 75 GPa, respectively, in the Rigaku PDXL 2.7 software (Rigaku, Wilmington, MA, USA) for residual stress calculation. A mention must be made regarding these values, as they are both approximate values and may not represent the true value of the material due to the anisotropy of AM materials [62]. Furthermore, L–PBF Al alloys are known for having anisotropic properties due to the different metallographic phases and therefore, may not be constant Faces E and F were chosen for analysis, allowing to evaluate two distinct build planes: XY (parallel to layer deposition) and Z (perpendicular to layer deposition) planes, respectively. Fourthly, to evaluate sample microstructure, samples were cut, sanded, polished and etched as detailed in Table 4 and Table 5. A gradient exposure was achieved by submerging the part in a progressive manner in the etching solution, allowing to visualize the surface under different etched conditions. Microstructures were viewed using a FEI Quanta 400 FED Scanning Electron Microscope (SEM) (FEI, Hillsboro, OR, USA) at several magnifications, along with an Energy Dispersive X–Ray Spectroscopy (EDS) analysis in areas of interest. Lastly, sample hardness was measured on the cut/polished surfaces for both XY and Z build planes. Hardness testing followed the procedures underlined in the ASTM F3122-14 and ASTM E10-18 standards [63,64].

## 3. Results and Discussion

### 3.1. Weight & Relative Density 

**Figure 3:**
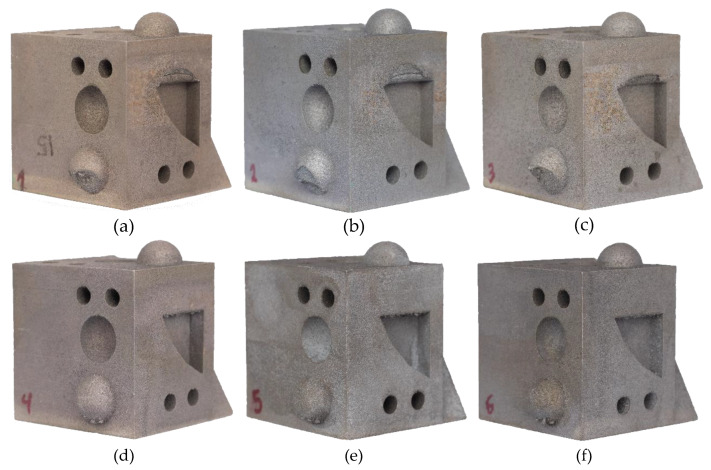
Final part examples from each sample set, showing the effects of using build support material on overhang features; (**a**) example from Sample_Set_01, built with a chessboard strategy, 15 µm layer and no support material; (**b**) example from Sample_Set_02, built with a continuous strategy, 15 µm layer and no support material; (**c**) example from Sample_Set_03, built with a chessboard strategy, 20 µm layer and no support material; (**d**) example from Sample_Set_04, built with a chessboard strategy, 15 µm layer and support material; (**e**) example from Sample_Set_05, built with a stripes (45°) strategy, 15 µm layer and support material; (**f**) example from Sample_Set_06, built with a stripes (67°) strategy, 15 µm layer and support material.

Table 6 presents the weight average and relative density of each sample set created with the parameters of Table 2. As can be seen, with the exception of Sample_Set_02, the samples are within less than one gram of each other, showing very good repeatability between production runs. Sample_Set_02 is relatively lower than other samples, showing that a continuous pattern strategy generated the lease dense samples. A similar outcome was also encountered by Aboulkhair, et al. [65], which found that when using a continuous unidirectional scan patter, along with scan speeds above 500 mm/s, part densities dropped below 95%. This is perhaps due to the rapid cooling rates seen by the strategies and lack of overlapping that leads to a higher degree of lack of fusion defects. Stripes and chessboard island strategies generate parts with similar weight and relative density, as the heat source is evenly concentrated in smaller areas and therefore allows for better fusion. These findings are in line with those of other authors, who have also found that chessboard strategies led to parts with higher density, a consequence of eliminating the majority of lack of fusion defects [66]. The use of supports structures does not have a significant influence on part density, as can be seen by comparing Sample_Set_01 with Sample_Set_04. However, some variation can be seen when comparing the groups of samples with and without support structures. Also, print time and energy per sample present no influence as samples set 1, 2 and 3 were subjected to different print times than 4, 5 and 6. A higher layer thickness of 20 µm also does not impact much these results, as seen by Sample_Set_03. An interesting aspect that can be noted is that all samples weighted below the expected theoretical weight and the 99% relative density mark was not achieved, a mark that has been achieved in other studies [65,67]. This is due to two main reasons, the existence of pores (mainly due to the lack of fusion) and the lack of material on overhang features. As can be seen Figure 3, sample sets 1, 2 and 3 all present defects/lack of material on overhanging features (defects on half dome of Face F and the top of the indenting triangle in Face D), which is due to the lack of support structures during manufacturing.

### 3.2. Residual Stress 

Although all samples were submitted to a heat treatment after production, residual stress was still present on all measured surfaces. Table 7 shows the encountered residual stress values for two distinct sample faces. With the exception of Sample_Set_05, all other samples showed higher residual stress values on Face F (left vertical face) than on Face E (top horizontal face), indicating that the Z plane is subjected to higher stress conditions. Sample_Set_04 presented the lowest residual stress values for both planes, indicating that the use of the manufacturer’s recommended settings (a chessboard strategy alongside with the use of support structures) generated lower stress values deduced when comparing this sample with Sample_Set_01. Indeed, the chessboard strategy has shown in other studies to generate lower residual stress, attributed to the shorter laser scan line lengths [66,68]. The use of support material has also shown to help conduct more heat and lower stress, as concluded by Salmi et al. [39]. The stripes strategies follow similar short laser line path lengths, in a continuous method instead of isolated islands. A study by Kruth, et al. [69] showed that continuous and directional strategies showed higher residual stresses than those seen by island strategies. Stripes strategies are performed in a sweeping column fashion, creating stripes/columns of solidified material. The orientation of the stripes is then rotated a specific angle (in the case of this study either 45° for Sample_Set_05 or 67° for Sample_Set_06), depositing the subsequent layer. The use of a 45° stripes strategy returned the highest registered stress values, seen on face E of Sample_Set_05, showing a high influence of this strategy on the XY plane layer, while the Z plane shows a stress behavior analogous to the other samples. It can be speculated that the origin for such large residual stress values can be a consequence of the chosen layer rotation angle, which when set at 45° (1/8 of 360°), facilitates layer orientation repetition every 8 layers, forming a specific pattern. Considering that this part had an upwards of 2300 layers, this repetition of layer orientation allows for stresses to build up in specific areas and never be offset by differently orientated layers. Sample_Set_06, using the stripes 67° strategy generated the second lowest residual stress value on the XY plane, further supporting the previous speculation as, a 67° of rotation at every layer avoids layer orientation repetition thus, allowing subsequent layers to rearrange previous surface stress/tensions. A literature review, performed by Kempf and Hilgenberg [70], showed that the mechanical properties, such as ultimate tensile strength, of samples produced with stripes strategies seems to marginally outperform chessboard strategy samples, in the as-built state. However, this trend is no longer present after heat treatments are applied. Indeed, residual stresses impact mechanical properties, especially in anisotropic materials where no preferential stress orientation is present, therefore, minimizing residual stress is advantageous for part strength and durability. When comparing Sample_Set_01 with Sample_Set_03, it is clear that larger layer thicknesses lead to higher residual stress, in either plane, being significantly more influential on Z plane faces. In relation to the total energy input per volume of material, it can be seen that printing time and input energy do not have an effect on residual stress, as there is a large variation among the samples produced in the same jobs. When using the manufacturer’s recommended process settings for layer thickness, scanning speed and laser power, the scanning strategies tested (chessboard (island), continuous and 67° stripes) promote very similar results, showing low influence on residual stress while 45° stripes promote higher stress levels on top faces (parallel to build direction). 

### 3.3. Roughness

For this work, it was established that lower surface roughness values were considered desirable, as this usually leads to better surface finishes and higher levels of detail. Table 8 shows the values measured on the different areas of the part (top and front face, as well as the 30°, 45° and 60° angled ramps) organized in ascending order of average roughness (R_a_). The absence of measured values for certain sample faces signifies that the maximum roughness depth ($\bar{R_{\max}}$) in these areas largely exceeded the 100 µm pick up range of the measuring equipment, indicating an irregular surface with larger peaks, valleys or both. Therefore, it can be said that the left side face (vertical face) returned the most consistent values, being able to provide readings on all samples. Roughly speaking, it can also be said that the average Ra range was between 8–12 µm, with small variation between samples, being equivalent to machined surfaces achieved via sawing, drilling, milling, turning, casting, or surfaces achieved via sand casting or forging [71]. Surface roughness, as well as surface defects, can also be influenced by chamber part placement with factors such as gas flow, weld pool gas emission, spattering and re-coating angles being influential [72]. To ensure low influence, parts were equally positioned on the build plate, at a 5°angle regarding the re-coater thus, ensuring low re-coater contact length. This value is based on the study of Moylan, et al. [73], which states that the contact length between the re-coater blade and faces parallel to the blade should be minimized to reduce forces.

#### 3.3.1. Top Face

When viewing Table 8, it is possible to state that the lowest achieved R_a_ values (for the top face) were produced by Sample_Set_04, built using the manufacture’s recommended settings, alongside a chessboard island strategy and with the use of support structures, being similar to sand casted, sawed and forged surfaces. This sample set shows a significant variation between R_z_ and R_max_ values, allowing to infer that the measured surfaces show considerable isolated defects and unevenness. When comparing with results achieved from other authors, it is possible to view that the R_a_ roughness is somewhat higher than desired. For instance, other authors have achieved R_a_ values as low as 4.5 µm in as built samples [47,50,74]. Viewing Sample_Set_01 (which shows a 12.2% increase in R_a_) it is possible to state that the absence of build supports led to a 12.2% increase in average roughness, with similar increases in terms of R_z_ and R_max_, showing a beneficial effect of using build supports. Sample_Set_02, using a continuous build strategy and no supports follows a similar behavior, showing a 20% increase in Ra and Rz. The remaining samples were unable to return values (R_max_ possibly surpassed the 100 µm threshold limit) so that 20 µm layer heights, coupled with scanning speeds of 480 mm/s, as well as stripe pattern build strategies, can be said to promote higher roughness top surfaces.

#### 3.3.2. Front Face

When measuring the front face of samples, as it was built perpendicular to layer deposition, the contact stylus was able to evaluate the surface defects created by the transitions between different layers. This face was the only one that allowed to return values on all tested samples, meaning that all R_z_ were below 100 µm. Sample_Set_01 returned the lowest R_a_ of 9.787 µm, being followed by Sample_Set_06 and Sample_Set_04 with similar values. These values are still considered low, however, some authors have also achieved Ra values lower than 6 µm on front and side faces [74]. Contrary to the measurements taken on the top face, the front face generated smoother surfaces when no build supports were employed (Sample_Set_01 and Sample_Set_04). This is consequence of the fact that support material was present on the perpendicular and adjacent cube faces to where the roughness measurements were performed, which most likely generated some degree of distortion during manufacturing. Nevertheless, regarding R_a_, the difference between samples is of 10% or less, showing consistent results. The remaining sample sets returned consistent but slightly higher values, ranging between 12.073–12.451 µm, being equivalent to forged, planned, sawed and sand casted surfaces. Regarding the variation between R_z_ and R_max_ results, it is possible to view that these results are relatively close to each other, showing that the surface is even, without having any noticeable large peaks or valleys. The smoothest surface was generated by Sample_Set_01, which shows the smallest variation between R_z_ and R_max_ values. Building strategies, scanning speed and layer thickness seems to be of low influence on these surfaces.

#### 3.3.3. Angled Ramps

When glancing over Table 8, it is easy perceived that the 30° was capable of returning the highest number measurements (5 out of 6), however, the smoothest surface was generated by Sample_Set_01 on a 45° ramp which is somewhat unexpected. The expected roughness on inclined ramps is impacted by layer height, being that lower angles should show a higher stair effect than steeper angles. This low roughness encountered on the 45° angle can be speculated to have a low staircase/step effect due to a surface fusion anomaly, which leads to a smoother surface. Calignano [75] argues that the roughness on inclined surfaces is governed by process parameters, which can lead to an oversized melt pool, causing adjacent powder particles to stick the periphery of the melt pool, eliminating the staircase effect completely. Nevertheless, this value should not be considered for comparative results, and should be considered an anomaly, even though measurements and standard deviations are consistent. With respect to the 30° ramp, layer thickness seems to have the most influence on surface roughness as Sample_Set_03, with the larger 20 µm layers height, were unable to return values. This is in line with what would be expected, considering the layering staircase effect. The use of support structures shows no influence on surface roughness however, build pattern clearly has, being that continuous and chessboard island strategies are more advantageous. Sample_Set_02 returned the surfaces with the lowest R_a_, R_z_ and R_max_ values, being also the sample with the lowest variation between R_z_ and R_max_ among the 30° group, showing an even surface. When addressing the results of the 45° ramps, the lowest R_a_ value average of all measurements can be found on Sample_Set_01, with an Ra of 8.770 µm, this sample also shows a low variation between R_z_ and R_max_. The use of support material led to an increase in R_a_, shown by comparing Sample_Set_04 with Sample_Set_01. As previously seen, higher layer thicknesses did not return values, while chessboard island and continuous pattern strategies generated smoother surfaces than the rest strategies. The 60° ramp was the only surface to be able to return a value for the 20 µm layer thickness however, it represents the highest R_a_ registered in all measurements of 14.00 µm, as well as having the largest variation between R_z_ and R_max_, showing an irregular surface. Samplet_Set_06, Sample_Set_01 and Sample_02 show very similar results between them, showing that at this level of inclination, pattern strategy has low influence, while build support seem to be more influential. Also, these sample sets were the only ones to return values on all 30°, 45°and 60° surfaces. However, there is no apparent trend between them to how angle change affects surface roughness, being that each sample set shows a different trend. It can also be stated that the manufacturer’s recommended settings (represented by Sample_Set_01) were able to return values on all tested surfaces, showing that, in relation to surface roughness, these parameters are indeed a good middle ground to manufacture parts. 

### 3.4. Hardness 

Table 9 shows the measured hardness values, in descending order, registered on two distinct surfaces of the sample sets. Standard deviation of values is low, with the overall deviation average being around 1% and the worst around 3.8%. The XY plane represents a section perpendicular to the build direction while the Z plane presents a parallel section to the build direction. Hardness was measured on the cross section of cut and polished samples, targeting mainly the core area of the sample. When viewing the results, no significant difference could be found between the hardness of both planes, affirming that hardness is not influenced by build direction. However, hardness is influenced by the different machine parameters and pattern strategies that were tested. Manufactured recommended settings (Sample_Set_01 and Sample_Set_04) returned the highest hardness results, with very low variation between them. Pattern strategy seems to be the factor that most influences hardness, showing that a continuous pattern strategy promotes the lowest hardness surfaces. Layer thickness and slower scanning speeds were also influential as shown by Sample_Set_02, generating the second lowest hardness value. Stripes pattern strategies generated intermediate surface hardness values. Factors, such as the presence of defects and metallic microstructures can have high influence on mechanical properties. The presence of defects, such as pores or inclusions, or the existence of softer microstructures, can lead to a lower surface hardness, therefore, it is essential to minimize the appearance of these phenomenon. Nevertheless, a study conducted by Kempf and Hilgenberg [70] demonstrated that the impact of porosity on mechanical properties was not as profound as it may seemed, being instead attributed to grain and sub-cell boundaries sizes. This study also compared the hardness between samples built using chessboard and stripes strategies, but showed no particular differences between strategies for both, as-built and heat-treated samples. By using larger layer thickness, or choosing pattern strategies that promote higher cooling rates (such as continuous strategies), the likelihood of the appearance of defects is larger. The use of support structures may also contribute to lower cooling rates, due to the higher thermal mass of the part and heat transfer between the heated build plate and the part, thus, minimizing the appearance of defects.

### 3.5. Microstructure

**Figure 4:**
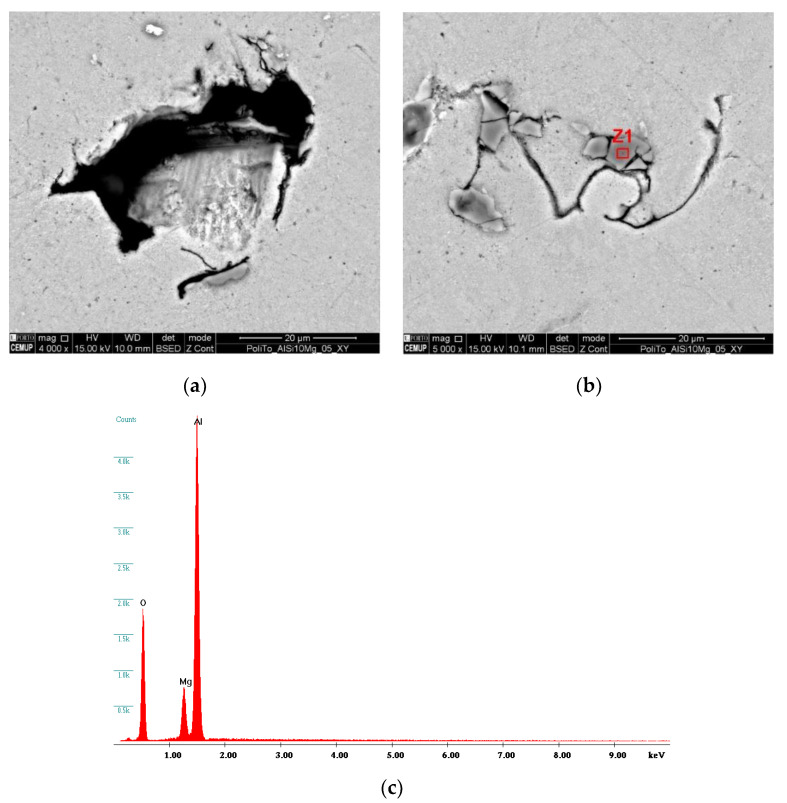
Encountered Defects on Alsi10mg Parts during SEM Analysis; (**a**) Lack of Fusion Pore; (**b**) Identification of Inclusion Marked as Z1; (**c**) analysis of Z1 Inclusion with EDS Equipment, Consistent with an Al–Mg Oxide.

**Figure 5:**
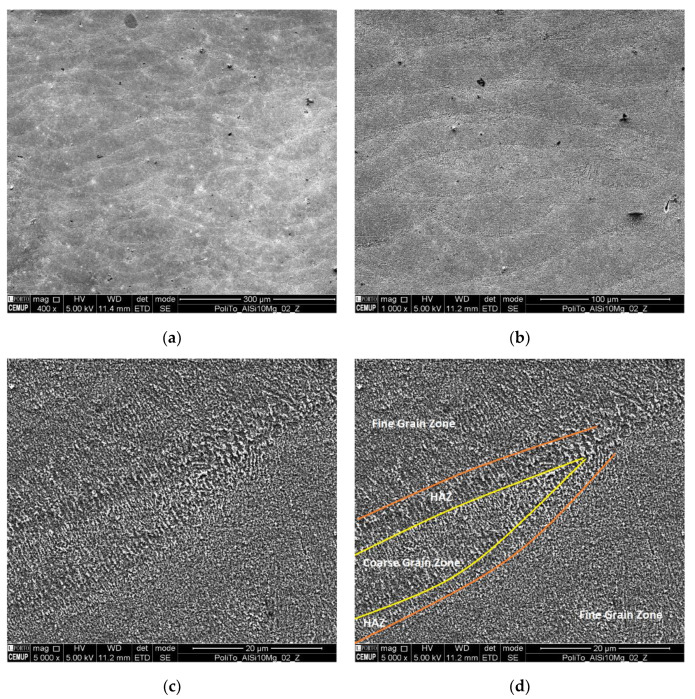
Microstructural Images Provided via SEM Analysis of Z Plane of Sample; (**a**) Overview of an Area at 400× Magnification, with Visual Building Up of Layers; (**b**) Exact Same Area at 1000× Magnification; (**c**) 5000× magnification close up; (**d**) identification of morphological areas seen in Figure 5c.

Samples were evaluated under a SEM equipment, allowing to visualize the presence of several surface defects, such as oxides inclusions, lack of fusion and pores. Figure 4 shows examples of the defects that were encountered. Figure 4a shows a pore that was generated from a lack of fusion, easily characterized by the presence of its irregular and jagged edges. Pores originating from entrapped gases are usually smaller and spherically shaped with round soft edges [27]. A lack of fusion defects, which are difficult to avoid, are mostly related to process parameters. Even though, AlSi10Mg parts with a build density of 99% in theory are possible, the process parameters necessary to achieve such a degree of density quality can be inefficient, as they are costly and have slow production times. Therefore, a balance between production cost/speed and part density must be set. Nevertheless, part density of AM parts remains competitive and can even be superior to conventional casting of Al–Si–Mg alloys, which is also prone to the appearance of pores, being able to reach, in some cases, pores with diameters of up to 2 mm [76]. Furthermore, when viewing Figure 5a,b, which was the common scenario across all sample, an absence of defects can be seen, as well as the different layers and respective thicknesses. This fact further fundaments that the variation in part density is also influenced by geometrical reproduction variation, such as poor reproduction of overhang features, due to the lack of support structures. Figure 4b highlights the presence of some darker areas, marked as Z1, deemed of interest and investigated with EDS analysis to better understand the elemental composition of this area. This analysis can be seen in Figure 4c, showing that this area is composed mainly of Al, Mg and O, being consistent with Al–Mg oxides found with these alloys. The origin of these oxides has been discussed in [22,77], concluding that they appear due to the oxidation of the metallic vapors that are released from the melt pool during material fusion. While Si has a low vapor pressure, Mg and Al have a high vapor pressure, becoming preferential to oxidation by the residual O present in the chamber [22].

Figure 5 shows an area in the Z plane, where the etching solution was able to promote visible results, being able to showcase the build direction and layer buildup, as shown in Figure 5a. Also, surface contamination can be noticed in both Figure 5a,b, being represented by several dark spots and scratches, which originated from the polishing procedure. These contaminations are consequence of the material’s low hardness and polished surface geometry (large flat surface area), which made polishing the surface more challenging. Upon a closer analysis, depicted in Figure 5c, grain morphology, size and boundaries can be differentiated. Figure 5d illustrates further these areas, distinguishing areas of finer grains, coarser grains and heat affected zones (HAZ). This microstructural distribution and morphology has also been observed by other researchers, showing similar results in [78] and [29].

**Figure 6:**
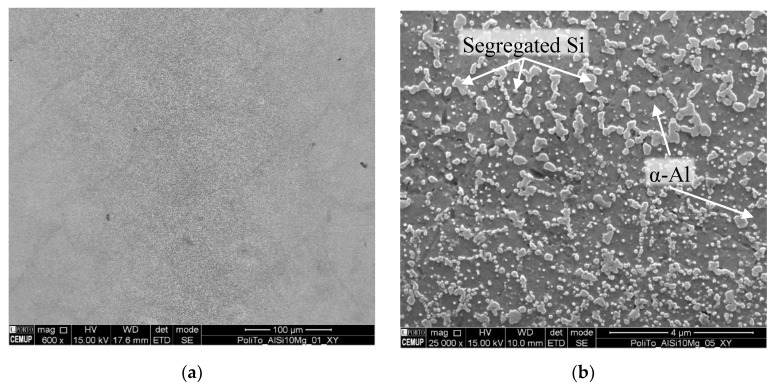
SEM Image of XY Plane of Sample; (**a**) General Overview of Area at 600× Magnification; (**b**) 25000× Magnified Areas, Illustrating Precipitated α–Al Dendrites Surrounded by Segregated Si.

The solidified microstructure of AlSi10Mg is mostly comprised of precipitated α–Al dendrites surrounded with segregated Si [29]. Figure 6 shows an example of this type of structure, which can be of different size depending on the temperatures and cooling speeds achieved during the production process, as shown in Figure 5d. The finer grain areas are made up of small α–Al cellular dendrites which are created during powder fusion or material re-melting during laser overlapping. These structures were originated due to the high temperatures and rapid cooling speeds seen during the process, with material melting and solidifying very rapidly, essentially hindering the growth of dendrites. Coarser areas, with larger equiaxed α–Al dendrites, are a consequence of areas that did not suffer any re-melting during laser passes but, achieved a semi-fused state, having been exposed to sub-fusion temperature, allowing for a larger and directional grain growth. The HAZ area was exposed to lower temperatures, simulating a heat treatment, allowing for the formation and growth of Si particles [30,78].

Takata, et al. [79], Fiocchi, Tuissi, Bassani and Biffi [42] and Lv, et al. [80] are examples of authors that have researched into the heat treatability of AlSi10Mg alloys, having observed that heat treatments in the 300 °C temperature range, or higher, lead to Si particle segregation from the supersaturated matrix in uniformly distributed manner throughout the α–Al matrix, as seen in Figure 6b, in the form a granular small particles. Also, a fading of layer and melt pool tracks begins to occur, generating a more uniform structure and mechanical properties. 

## 4. Conclusions

After viewing the results as a whole, it is possible to state that the samples that achieved the overall best results belong to the groups of Sample_Set_01 and Sample_Set_04, indicating that a layer thickness of 15 µm, scanning speeds of 650 mm/s and a chessboard pattern strategy exhibit improved outcomes when compared to the remaining results. Although the part has a large base, the use of support structures generated parts with lower residual stress, possibly due to the better cooling capabilities that these structures provided, allowing heat transfer to the base plate. However, no group of samples was able to excel at all tested aspects at once, indicating that parameters and strategies must be selected based on the objectives of the final results. In a broad sense, the following can be stated:

- continuous pattern strategies seem to generate less denser parts, this is mainly due to the incorporation of defects such as lack of fusion/pores, associated with this strategy [66];
- all samples weighted below the theoretical weight, mainly due to the inclusion of pores by a lack of fusion;
- chessboard and stripes 67° pattern strategies seem to promote the best overall results, as the samples produced by these strategies are always among the best three in the tested categories;
- residual stress is higher on vertical faces than horizontal faces;
- the use of chessboard strategies along with build support structures generated the lowest residual stress values;
- R_a_ surface roughness ranges between 8–12 µm, being equivalent to certain machined surfaces (originating from sawing, drilling, milling and turning processes), casted surfaces and forged surfaces;
- build orientation does not influence surface hardness;
- printing time and average energy input per sample do not influence the measured parameters;
- hardness is influenced by machine parameters and highly affected by the pattern strategy that is selected;
- chessboard strategy presents the highest results of all tested, achieving in this case a hardness ranging between 113–119 HBW for both XY and Z planes;
- the main defects encountered were pores originating from the lack of fusion and inclusions of Al-Mg oxides;
- the observed microstructure is composed of precipitated α–Al dendrites bordered with segregated Si;
- the grain size of the α–Al dendrites varies depending on their location regarding HAZ and fusion zone areas.

## Figures and Tables

**Table 1:** AlSi10Mg chemical composition and mechanical properties (post heat treatment) [55,56,57,58,59].

**Chemical Composition**			
**Component**	**wt%**	**Component**	**wt%**
Al	Balance	Cu	0–0.10
Si	9.1–11.0	Zn	0–0.10
Mg	0.20–0.45	C	0–0.05
Fe	0–0.55	Ni	0–0.05
Mn	0–0.45	Pb	0–0.05
Ti	0–0.15	Sn	0–0.05
**Mechanical Properties ^1^**			
	90° (horizontal)	45° (polar angle)	0° (upright)
Tensile Strength (MPa)	329 ± 4	346 ± 3	344 ± 2
Yield Strength (MPa)	211 ± 4	215 ± 3	205 ± 3
Elongation (%)	9 ± 1	7 ± 1	6 ± 1
Density ^2^ (kg/m^3^)	2680		
Hardness (HBW) ^2,3^	120 ± 5		
Young’s Modulus (GPa)	≈75		
Poisson’s Ratio	0.33		
Fatigue Strength ^2^ (MPa)	97 ± 7		
Thermal Conductivity ^2^ (W/m⋅K)	120–180		
Thermal Expansion ^2^ (10–6/K)	20		

^1^ – According to the DIN EN 50125; ^2^ – Build orientation not specified; ^3^ – According to DIN EN ISO 6506–1.

**Table 2:** Selected AM Process Parameters.

Reference	Material	Pattern Strategy	Layer (μm)	Scan Speed (mm/s)	Laser Power (W)	Build Support	HT	Avg. Print Time ^1^	Avg. Enerygy (kWh) ^1^
Sample_Set_01	AlSi10Mg	Chessboard (Island)	15	650	95	No	1 h @ 310 °C	14 h 59 min ^2^	10.890
Sample_Set_02	AlSi10Mg	Continuous	15	650	95	No	1 h @ 310 °C	14 h 59 min ^2^	10.890
Sample_Set_03	AlSi10Mg	Chessboard (Island)	20	480	95	No	1 h @ 310 °C	14 h 59 min ^2^	10.890
Sample_Set_04	AlSi10Mg	Chessboard (Island)	15	650	95	Yes	1 h @ 310 °C	13 h 46 min ^3^	12.176
Sample_Set_05	AlSi10Mg	STRIPES (45°)	15	650	95	Yes	1 h @ 310 °C	13 h 46 min ^3^	12.176
Sample_Set_06	AlSi10Mg	STRIPES (67°)	15	650	95	Yes	1 h @ 310 °C	13 h 46 min ^3^	11.920

^1^ – per sample calculated from overall print time; ^2^ – printed in the same jobs to optimize print area and cost (total layer count of 4300); ^3^ – printed in the same jobs to optimize print area and cost (total layer count 2867).

**Table 3:** Sample evaluation equipment and parameters.

Property to Evaluate	Equipment	Parameters	
Roughness	Perthometer M2	Lt (mm)	5.60 [N = 5]
		Lm (mm)	4
		Lc (µm)	0.8
		Vt (mm/s)	0.50
		Points	11200
		Pick-up	NHT 6–100
		Pick-up range (µm)	100
		Pick-up contact point (mm)	0.8
Residual Stress	Rigaku Smartlab^®^	Radiation Source	CuK_α_
		Software	PDXL 2.7
Microstructure	FEI Quanta 400 FEG (Field Emission Gun) SEM (Scanning Electron Microscopy)	Equipped with EDAX EDS (Energy Dispersive Spectroscopy) analyser	
Hardness	EMCO TEST M4U 025 G3	Standard	ASTM E10
		Hardness Abbreviation	HBW 2.5/62.5
		Indenter Ball Ø(mm)	2.5
Weight	Denver Instrument APX–200 Scale	Capacity (g)	200
		Readability (mg)	0.1

**Table 4:** Surface Preparation Steps for Microstructure Observation.

Step.	Equipment	Parameters	Consumables
Cutting	Presi Mecatome T300	RPM: 3200 Coolant: On Mode: Manual	Cutting Discs: - Presi 01023 (SiC abrasive for non-ferrous materials).
Sanding	Rotopol-1	RPM: 150 Coolant: On	500, 800 and 1200 grit sandpaper discs.
Polishing	Rotopol-1	RPM: 150	Felt polishing pads, polishing lubricant, 3 µm and 1 µm diamond cutting compound.

**Table 5:** Etching solution and exposure times.

Etching Solution	Exposure
Keller’s Reagent (2 mL HF (48%), 3 mL HCl (34%), 5 mL HNO_3_ (70%), 190 mL H_2_O	8–20 s (submerged progressively creating a gradient etched surface)

**Table 6:** Average Weight and Standard Deviation for Each Sample Set.

Reference	Average Weight (g)	Stand. Dev.	Relative Density (%) ^1^	Pattern Strategy	Layer Thickness (μm)	Scanning Speed (mm/s)	Build Support
Sample_Set_01	94.00	0.09250	98.16	Chessboard	15	650	No
Sample_Set_02	91.63	0.03759	95.69	Continuous	15	650	No
Sample_Set_03	93.12	0.01136	97.24	Chessboard	20	480	No
Sample_Set_04	93.92	0.01258	98.08	Chessboard	15	650	Yes
Sample_Set_05	93.45	0.05052	97.59	STRIPES (45°)	15	650	Yes
Sample_Set_06	93.97	0.05745	98.13	STRIPES (67°)	15	650	Yes
Theoretical	95.76^1^	-	-	-	-	-	-

^1^ – determined by the weight difference between real and 3D scanned sample weights.

**Table 7:** Residual stress values of faces E and F found at approximately *2θ* = 78°.

Reference	Face E ^1^ (MPa)	Stand. Dev.	Face F ^2^ (MPa)	Stand. Dev.	Pattern Strategy	Layer Thickness (μm)	Scanning Speed (mm/s)	Build Support
Sample_Set_01 ^3,5^	256.1	28.84	318.3	21.43	Chessboard	15	650	No
Sample_Set_02 ^3,5^	256.2	47.73	300.2	50.38	Continuous	15	650	No
Sample_Set_03 ^3,5^	363.9	48.10	435.1	53.93	Chessboard	20	480	No
Sample_Set_04 ^4,5^	194.6	19.95	239.7	25.64	Chessboard	15	650	Yes
Sample_Set_05 ^4,5^	646.4	100.4	314.3	42.70	STRIPES (45°)	15	650	Yes
Sample_Set_06 ^4,5^	205.4	16.02	304.0	44.61	STRIPES (67°)	15	650	Yes

^1^ – XY Plane, parallel to layer deposition; ^2^ – Z Plane, perpendicular to layer deposition; ^3^,^4^ – produced in the same print job respectfully, ^5^ – heat treated for 1 h at 350 °C.

**Table 8:** Returned Roughness Values for Faces E and F, as well as 30°, 45° and 60° Angled Ramps.

Sample Face	Reference	$\bar{R_{a}}\;(\mu m)$	Stand. Dev	%Δ (%)	$\bar{R_{z}}\;(\mu m)$	Stand. Dev	%Δ (%)	$\bar{R_{\max}}\;(\mu m)$	Stand. Dev	%Δ (%)
Face E (Top)	Sample_Set_04	10.64	1.516	0.000	52.79	5.490	0.000	74.77	9.457	0.000
	Sample_Set_01	11.94	0.094	12.20	58.51	0.625	10.85	83.33	8.505	11.45
	Sample_Set_02	12.85	0.995	20.76	63.38	5.507	20.06	80.62	8.190	7.824
	Sample_Set_03	-	-	-	-	-	-	-	-	-
	Sample_Set_05	-	-	-	-	-	-	-	-	-
	Sample_Set_06	-	-	-	-	-	-	-	-	-
Face F (Left)	Sample_Set_01	9.787	0.746	0.000	54.25	3.066	0.000	63.96	6.853	0.000
	Sample_Set_06	10.55	0.285	7.752	57.16	1.483	5.361	75.58	9.727	18.17
	Sample_Set_04	10.75	2.281	9.863	55.31	7.062	1.963	67.06	2.998	4.847
	Sample_Set_03	12.07	2.460	23.36	59.72	7.644	10.09	75.93	12.47	18.71
	Sample_Set_05	12.27	1.699	25.35	62.46	5.651	15.14	75.57	10.27	18.15
	Sample_Set_02	12.45	0.558	27.22	62.79	3.523	15.75	77.06	14.59	20.48
30° Ramp	Sample_Set_02	9.329	0.039	0.000	48.26	1.563	0.000	55.79	1.563	0.000
	Sample_Set_04	10.18	0.812	9.083	52.21	4.210	8.187	66.10	4.210	8.187
	Sample_Set_01	11.28	0.136	20.87	57.09	0.410	18.30	73.43	0.406	18.30
	Sample_Set_06	11.93	1.004	27.86	64.37	1.874	33.37	83.27	1.874	33.37
	Sample_Set_05	12.35	0.2411	32.43	63.02	2.040	30.57	93.42	2.040	30.57
	Sample_Set_03	-	-	-	-	-	-	-	-	-
45° Ramp	Sample_Set_01	8.770	0.138	0.000	51.94	1.490	0.000	62.45	2.838	0.000
	Sample_Set_02	11.75	0.484	33.99	61.78	0.989	18.94	80.45	7.076	28.82
	Sample_Set_04	11.79	0.263	34.43	68.13	2.146	31.18	79.21	2.786	26.84
	Sample_Set_06	12.18	1.169	38.82	60.84	5.427	17.14	79.17	13.13	26.76
	Sample_Set_03	-	-	-	-	-	-	-	-	-
	Sample_Set_05	-	-	-	-	-	-	-	-	-
60° Ramp	Sample_Set_06	10.47	0.042	0.000	55.08	2.655	0.000	73.16	2.112	0.000
	Sample_Set_01	10.60	0.051	1.232	58.64	2.267	6.473	73.83	5.266	0.916
	Sample_Set_02	10.83	1.971	3.480	58.72	10.19	6.614	80.69	6.480	10.29
	Sample_Set_03	14.00	0.308	33.71	79.10	1.494	43.63	103.0	1.753	40.73
	Sample_Set_04	-	-	-	-	-	-	-	-	-
	Sample_Set_05	-	-	-	-	-	-	-	-	-

**Table 9:** Hardness Values Found on XY and Z Planes Measured on Cut/Polished Faces.

Planes	Reference	Hardness (HBW)	Stand. Dev	Pattern Strategy	Layer Thickness (μm)	Scanning Speed (mm/s)	Build Support
XY Plane	Sample_Set_01	119	1.550	Chessboard	15	650	No
	Sample_Set_04	117	1.472	Chessboard	15	650	Yes
	Sample_Set_06	95.6	0.736	Stripes (67°)	15	650	Yes
	Sample_Set_05	92.5	0.408	Stripes (45°)	15	650	Yes
	Sample_Set_03	89.1	0.761	Chessboard	20	480	No
	Sample_Set_02	87.5	0.797	Continuous	15	650	No
Z Plane	Sample_Set_01	117	0.894	Chessboard	15	650	No
	Sample_Set_04	113	0.816	Chessboard	15	650	Yes
	Sample_Set_06	96.3	0.703	Stripes (67°)	15	650	Yes
	Sample_Set_05	91.5	1.021	Stripes (45°)	15	650	Yes
	Sample_Set_03	90.9	1.160	Chessboard	20	480	No
	Sample_Set_02	86.7	3.311	Continuous	15	650	No
